# Genetic variations and microbiome of the poultry red mite *Dermanyssus gallinae*

**DOI:** 10.3389/fmicb.2022.1031535

**Published:** 2022-11-08

**Authors:** Yudai Nishide, Takafumi N. Sugimoto, Kenji Watanabe, Hiroshi Egami, Daisuke Kageyama

**Affiliations:** ^1^Institute of Agrobiological Sciences, National Agriculture and Food Research Organization (NARO), Tsukuba, Ibaraki, Japan; ^2^Research and Development Sector, SC Environmental Science Co., Ltd., Chuo-ku,Tokyo, Japan

**Keywords:** symbiont bacteria, *Bartonella*, *Rickettsiella*, *Cardinium*, *Wolbachia*

## Abstract

The poultry red mite *Dermanyssus gallinae* poses a significant threat to the health of hens and poultry production. A comprehensive understanding of *D. gallinae* is necessary to develop sustainable and efficacious control methods. Here we examined 144 *D. gallinae* collected from 18 poultry farms throughout the Japanese Archipelago for their genetic variations based on mitochondrial cytochrome c oxidase subunit I (COI) sequences, and microbiome variations based on amplicon sequencing of the 16S ribosomal RNA gene. According to COI sequencing, the Japanese samples were categorized into three haplogroups, which did not reflect the geographical distribution. Microbiome analyses found that the major bacteria associated with *D. gallinae* were *Bartonella*, *Cardinium*, *Wolbachia*, and *Tsukamurella*, with *Bartonella* being most predominant. Among 144 individual mites, all possessed one of the two major types of *Bartonella* (*Bartonella* sp. A), while 140 mites possessed the other type (*Bartonella* sp. B). The presence of the two strains of *Bartonella* was also confirmed by a single copy gene, *rpoB*. The presence of *Bartonella* in laid eggs suggested transovarial vertical transmission. Given that obligate blood-feeding arthropods generally require a supply of B vitamins from symbiotic bacteria, *Bartonella* may play an important role in mite survival. *Rickettsiella*, a major symbiont in European *D. gallinae* populations, and suggested to be an important symbiont by genomic data, was rarely found in Japanese populations. *Cardinium* detected from *D. gallinae* fell into a major clade found widely in arthropods, whereas *Wolbachia* detected in Japanese *D. gallinae* appear to be a new lineage, located at the base of *Wolbachia* phylogeny. Of the mitochondrial phylogeny, infection patterns of *Cardinium* and *Wolbachia* were strongly correlated, possibly suggesting one or both of the symbionts induce reproductive manipulations and increase spread in the host populations.

## Introduction

The poultry red mite *Dermanyssus gallinae* (Acari: Dermanyssidae), an obligate blood-feeding ectoparasite that feeds on avian blood, is globally distributed (Chauve, 1998; Sparagano et al., 2009; Wang et al., 2010), and is endemic in many commercial poultry farms, with 80%–90% of egg-laying facilities being infested (Sparagano et al., 2009; George et al., 2015). Once these mites invade a poultry house, their numbers can increase dramatically because typical conditions within poultry houses (high temperature and humidity) are ideal for *D. gallinae*. The densities of *D. gallinae* often reach up to 50,000 mites per bird. In extreme cases when densities reach 500,000 mites per bird, a hen can lose more than 3% of its blood volume every night (Kilpinen et al., 2005). Such heavy mite infestations seriously impact hen health and welfare, resulting in anemia and irritation, and can cause a 10-fold increase in hen mortality (Sigognault Flochlay et al., 2017). Predictably, *D. gallinae* causes a significant reduction in both egg quality and production (Kilpinen et al., 2005; Sparagano et al., 2014). In Europe, *D. gallinae* infestation costs the poultry industry over €231 million annually (Sigognault Flochlay et al., 2017). Furthermore, the prevalence of *D. gallinae* is expected to increase due to increasing acaricide resistance, climate change, and the lack of a sustainable and efficacious approach to control infestations (Chauve, 1998; Nordenfors et al., 2001). Increasing fundamental knowledge of *D. gallinae* can provide insights into new control methods.

Obligatory hematophagy, the practice of feeding exclusively on blood throughout all life stages, is found in a variety of arthropods. Because blood is nutritionally unbalanced, with high levels of protein, iron, and salt, but few carbohydrates, lipids, or vitamins, obligatorily hematophagous arthropods typically rely on symbiotic bacteria to obtain B vitamins (Husnik, 2018; Duron and Gottlieb, 2020). For example, tsetse flies *Glossina* spp. and the bed bug *Cimex lectularius* depend on their endosymbionts *Wigglesworthia glossinidia* and *Wolbachia* for their B vitamin supplies, respectively (Akman et al., 2002; Hosokawa et al., 2010; Michalkova et al., 2014; Nikoh et al., 2014; Moriyama et al., 2015). However, a controversy exists over vitamin-supplying symbiotic bacteria of the obligatory hematophagous *D. gallinae*. Initially, *Rickettsia* was suggested to be a symbiotic bacterium of *D. gallinae* in France using PCR amplification and fingerprinting methods (De Luna et al., 2009; Moro et al., 2009). According to Hubert et al. (2017), however, *Bartonella*-like bacteria rather than *Rickettsia* were considered as the mutualistic symbionts of *D. gallinae* because *Bartonella*-like bacteria was found in four of four sampling sites in Czechia, and in all stadia including eggs by amplicon sequencing. In contrast, another study showed that *Rickettsiella* was widespread in Europe and pseudogenized for many genes, including those involved in the amino acid synthesis pathway, but had an almost full set of genes for B vitamins biosynthesis (Price et al., 2021). In the aforementioned Czech study (Hubert et al., 2017), *Rickettsiella* was found in samples from only one of the four sites. Considering these discrepancies, this study investigated the genetic variations and microbiome variations of *D. galline* in Japanese poultry populations.

## Materials and methods

### Mite collection

**Figure 1:**
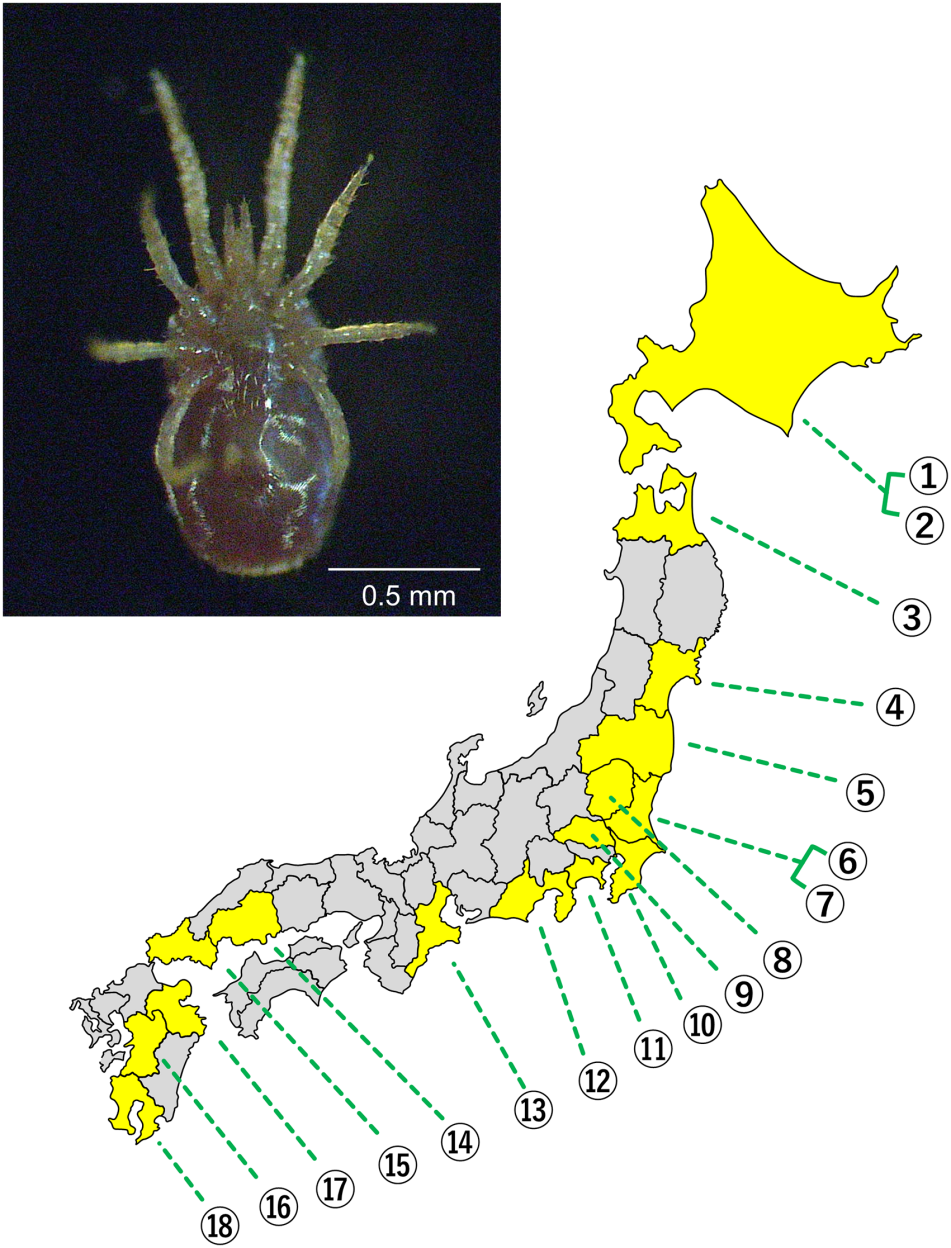
(Upper) Ventral view of adult *D. gallinae*. (Lower) A map of the Japanese archipelago. Prefectures where the mites were sampled are depicted in yellow. Each number represents a poultry farm. The exact location of each poultry farm is concealed.

In total, 144 individual *D*. *gallinae* mites collected from 18 poultry farms in 16 Japanese prefectures were brought to the laboratory alive and preserved in 99.5% ethanol at 4°C until DNA extraction was performed (Figure 1; Supplementary Table 1).

### DNA extraction, PCR, and cloning

After being crushed using an EOG-sterilized BioMasher II (Nippi, Inc., Tokyo, Japan), DNA was extracted from the whole body of each adult *D. gallinae* using a DNeasy Blood and Tissue Kit (Qiagen, N.V., Venlo, Netherlands) with 50 μl of EB buffer. The DNA samples were stored at −30°C until used. PCR was conducted for mitochondrial cytochrome *c* oxidase subunit I (COI) using KOD FX Neo (Toyobo Co. Ltd., Osaka, Japan), and the primers FCOIDG and RCOIDG designed for COI of *D. gallinae* (Øines and Brännström, 2011). For 16S ribosomal RNA sequencing of symbiont bacteria, PCR was conducted using Ex Taq HS (Takara Bio Inc., Kusatsu, Japan) and two sets of universal primers, 10F-1507R or 10FF-1515R. The PCR fragment was cloned using pGEM-T Easy Vector (Promega, Madison, WI, USA), Mighty mix for DNA ligation (Takara), and *Escherichia coli* DH5α competent cells (Takara). Plasmids were extracted using a QIAprep Spin miniprep kit (Qiagen), the purified plasmids were subjected to sequencing reactions using a BigDye Terminator v3.1 Cycle Sequencing Kit (Thermo Fisher Scientific, Waltham, MA, USA) and the sequencing primers M13F or M13RV in the flanking regions of the vector along with the bacterial universal primer 16SA2. The primer sequences are shown in Supplementary Table 2.

### Molecular phylogenetic analysis

The molecular phylogenetic analyses were conducted utilizing maximum-likelihood estimation methods using MEGA: Molecular Evolutionary Genetics Analysis, ver. 5.2 software (Tamura et al., 2011) with 1,000 bootstrap replications. The optimum model was selected through model tests to describe each phylogenetic tree. To analyze the genetic diversity of COI sequences, a network diagram of COI haplotypes was drawn using TCS 1.21 software (Templeton et al., 1992).

### Amplicon sequencing

To analyze the *D. gallinae* microbiome, hypervariable V3/V4 regions of the 16S rRNA gene were sequenced. The libraries created by using 2-step tailed PCR (Supplementary Table 2) were checked using a Synergy H1 (BioTek, Winooski, VT, USA) and a QuantiFluor dsDNA System (Promega), and the qualities were verified using a Fragment Analyzer (Agilent, Santa Clara, CA, USA) and a dsDNA 915 Reagent Kit (Agilent). These libraries were sequenced using a Miseq sequencer (Illumina, Inc., San Diego, CA USA), and the raw data were deposited in the GenBank sequence database (Accession No. DRR376882–DRR377025).

### Bioinformatic analysis of microbiota

The raw amplicon sequences were individually demultiplexed and converted to FASTQ files, which were then analyzed using the Quantitative Insights Into Microbial Ecology (QIIME2) v.2020.06 pipelines (Bolyen et al., 2019). Denoising and quality control, quality filtering, correction of errors in marginal sequences, removal of chimeric sequences, removal of singletons, joining of paired-end reads, and dereplication were conducted using the DADA2 plugin with the following option: -p-trim-left-f 10 -p-trim-left-r 10 -p-trunc-len-f 270 -p-trunc-len-r 270 (Callahan et al., 2016). The DADA2 algorithm was also used to cluster representative amplicon sequence variants and to provide count frequencies for each sample. Taxonomy classification was assigned to the representative amplicon sequence variants based on the Silva v.128 99% operational taxonomic units (OTU) reference sequences^1^, and the GreenGenes V.13_8 99% OTU reference sequences^2^ databases at 97% OTU level, trained using a Naïve Bayes classifier (classify-sklearn) and the q2-feature-classifier QIIME2 plugin (McDonald et al., 2012; Quast et al., 2013; Bokulich et al., 2018). The resulting relative abundance table of annotated amplicon sequence variants was exported and used to generate taxonomy bar plots in order to visualize the relative abundance of the microbiome using QIIME2 VIEW^3^.

### Analysis of *Bartonella* on *rpoB* sequences

Two distinct 16S rRNA gene sequences of *Bartonella* (*Bartonella* sp. A and *Bartonella* sp. B) may represent either the existence of distinct bacteria or the intragenomic operon copies of a single bacterium. To distinguish these possibilities, we performed PCR, cloning, and sequencing for the single copy gene *rpoB* from an individual (18_4) whose microbiome was occupied mostly with *Bartonella* sp. A and *Bartonella* sp. B with similar abundance. A primer set, Univ_rpoB_F_deg and Univ_rpoB_R_deg (Supplementary Table 2; Ogier et al., 2019), was used for PCR and the cloning and sequencing were performed as described above.

### Diagnostic PCR for *Rickettsiella*

PCR was conducted using KOD FX Neo or Ex Taq HS. The primers Rick-F and Rick-R were the same as those used in a previous study (Price et al., 2021). The amplicon was verified using 1.5% (w/v) agarose/TAE gel.

### Confirmation of bacteria in eggs

DNA was extracted from a pool of approximately 20 eggs freshly laid by *D. gallinae* females. The eggs were washed with 0.1% (w/v) benzalkonium chloride followed by two 5 min washes in 70% (v/v) ethanol, and were then subjected to DNA extraction and PCR using 27F-short and 1507R primers (Price et al., 2021). Cloning, plasmid extraction, and sequencing were performed as stated previously.

### Fluorescence *in situ* hybridization

Whole-mount fluorescent *in situ* hybridization (FISH) targeting bacterial 16S rRNA was performed using the mites collected from Aomori Prefecture following Koga et al. (2009). For the detection of bacterial 16S rRNA, we used the probe EUB338 (5′-GCT GCC TCC CGT AGG AGT-3′) (Amann et al., 1990) labeled with Alexa Fluor 555 at the 5′ terminus. The samples were incubated in hybridization buffer containing 50 nM probe. After being washed in PBST, host nuclear DNA was stained with 4.5 μM 4′,6-diamidino-2-phenylindole (DAPI; Thermo Fisher Scientific). Then, the samples were washed with PBST again, mounted in SlowFade Diamond Antifademountant (Thermo Fisher Scientific), and observed under a laser scanning confocal microscope (LSM 700; Carl Zeiss, Germany).

### Statistical analysis

In order to test whether the two bacteria, *Cardinium* and *Wolbachia*, share evolutionary trajectories in maternal lineages, the log marginal likelihood estimated on the COI tree with an assumed independent model was compared with that estimated with an assumed dependent model using BayesTraits V4.0.0.^4^ The log Bayes Factor (LBF), twice the difference between the two log marginal likelihoods, was used to interpret whether presence of the two bacteria were correlated (Gilks et al., 1995). LBF < 2 is considered weak evidence, 2 < LBF < 5 is considered positive evidence, 5 < LBF < 10 is considered strong evidence, and LBF >10 is considered very strong evidence.

## Results and discussion

### Mitochondrial COI sequences of *Dermanyssus gallinae*

**Figure 2:**
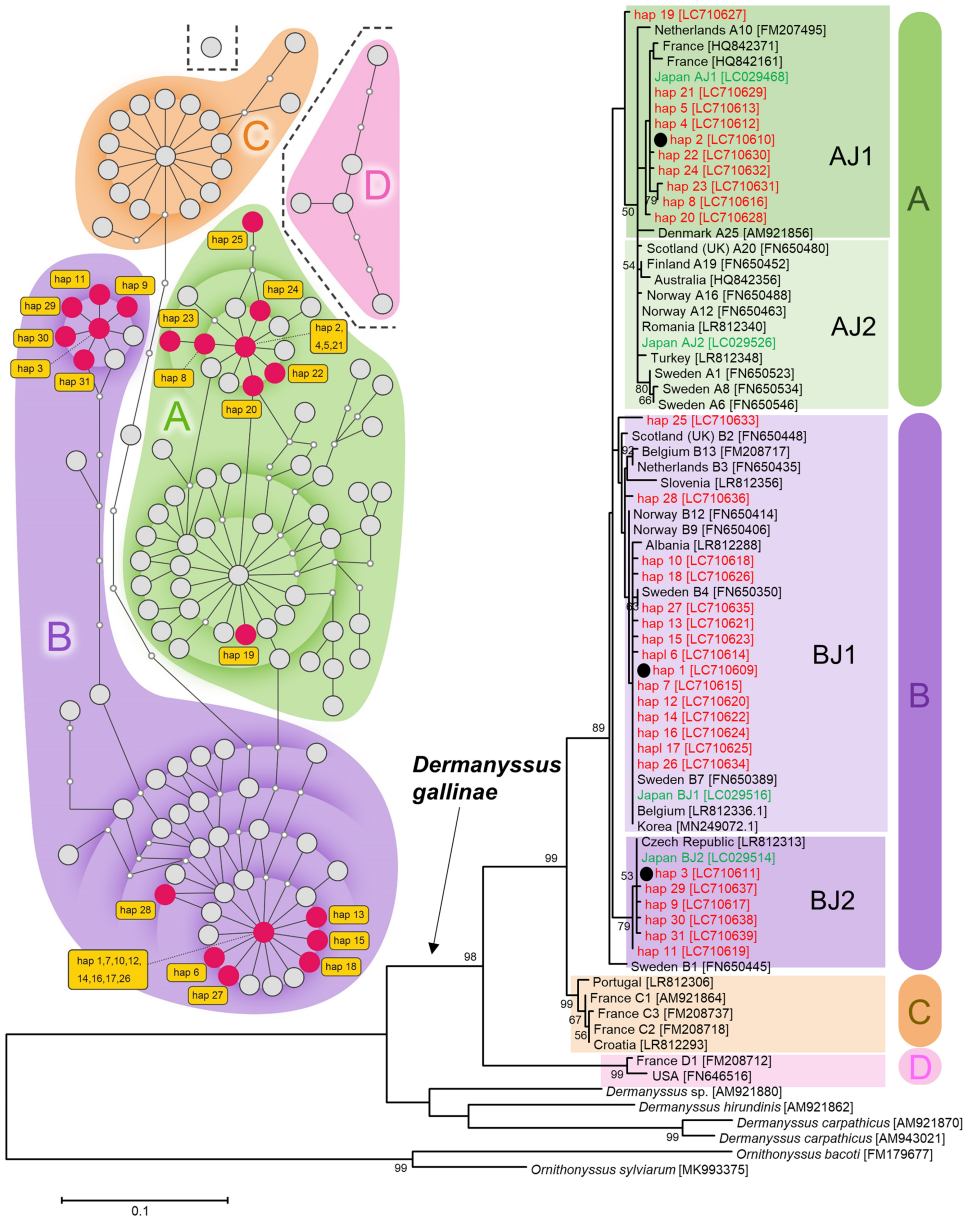
Mitochondrial DNA diversity of Japanese *D. gallinae* examined in this study among global samples of *D. gallinae*. *Left*: A TCS haplotype network based on COI sequences (314 bp; 122 OTU) of *D. gallinae*. *Right*: A maximum-likelihood phylogenetic tree (model T92 + G) based on COI sequences (469 bp; 75 OTU) of *D. gallinae* together with three other species of *Dermanyssus* and two species of *Ornithonyssus*. Based on the 469-bp sequences, 144 individual mites examined in this study fell into 31 haplotypes (hap 1–hap 31). Haplogroups **(A–D)** are shaded by respective colors. For the network, existing haplotypes are represented by large circles, and missing haplotypes are represented by small circles which are connected by branches representing 1-bp substitutions. Based on the 314-bp sequences, the 31 haplotypes were assigned into 21 haplotypes (indicated by red circles in the network). For the phylogenetic tree, the three most representative haplotypes (hap 1, hap 2, and hap 3) are indicated with black circles and their bootstrap probability is shown at each node. Nomenclature of haplotypes used in the previous studies (Øines and Brännström, 2011; Chu et al., 2015) are given following the country names. Haplotypes of Japanese samples are shown in red (this study) and green (Chu et al., 2015). Numbers in square brackets indicate accession numbers of NCBI. See Supplementary Table 3 for sample information.

By sequencing the mitochondrial COI region of 144 *D. gallinae* mites collected from 18 poultry farms (Figure 1), 31 haplotypes were categorized into three haplogroups AJ1, BJ1, and BJ2 according to previous studies (Øines and Brännström, 2011; Chu et al., 2015; Figure 2; Supplementary Figure 1; Supplementary Table 3). These haplogroups, however, do not necessarily reflect phylogenetically supported clades. The primary haplogroup BJ1 was closely related to several European populations (Norwegian, Swedish, Belgian) and a Korean population, whereas BJ2 was related to a Czech population (Figure 2; Supplementary Figure 2; Supplementary Table 4). These results may represent inter-country transport of contaminated material or infested birds as previously suggested (Øines and Brännström, 2011; Chu et al., 2015). AJ1 has only been found in the Japanese population, although it is relatively similar to the French population. AJ2 was found in Japan approximately 10 years ago (Chu et al., 2015), but was not found in the present study. Because multiple haplogroups were sometimes seen on a single poultry farm (Supplementary Table 3), invasions of *D. gallinae* are not likely to be very rare. In each poultry farm, chicken breeds are selected depending on their purpose (whether for eggs or meat), but mitochondrial haplogroups of *D. gallinae* were not associated with chicken breeds (purposes) (Supplementary Table 1). All 144 sample mites examined in the present study were identified as members of the well-supported clade of *D. gallinae*. Note that, as written below, *D. gallinae* possesses *Wolbachia* and *Cardinium*, which may confound the inference of an arthropod’s evolutionary history from mtDNA data because of maternal inheritance and linkage disequilibrium with mitochondria. Finally, the northern fowl mite, *Ornithonyssus sylviarum*, which resembles *D. gallinae* in size and appearance (Nakamae et al., 1997; Di Palma et al., 2012), is also known as a serious pest in poultry farms, but *O. sylviarum* was not found in the present samples.

### Microbiome of *Dermanyssus gallinae*

**Figure 3:**
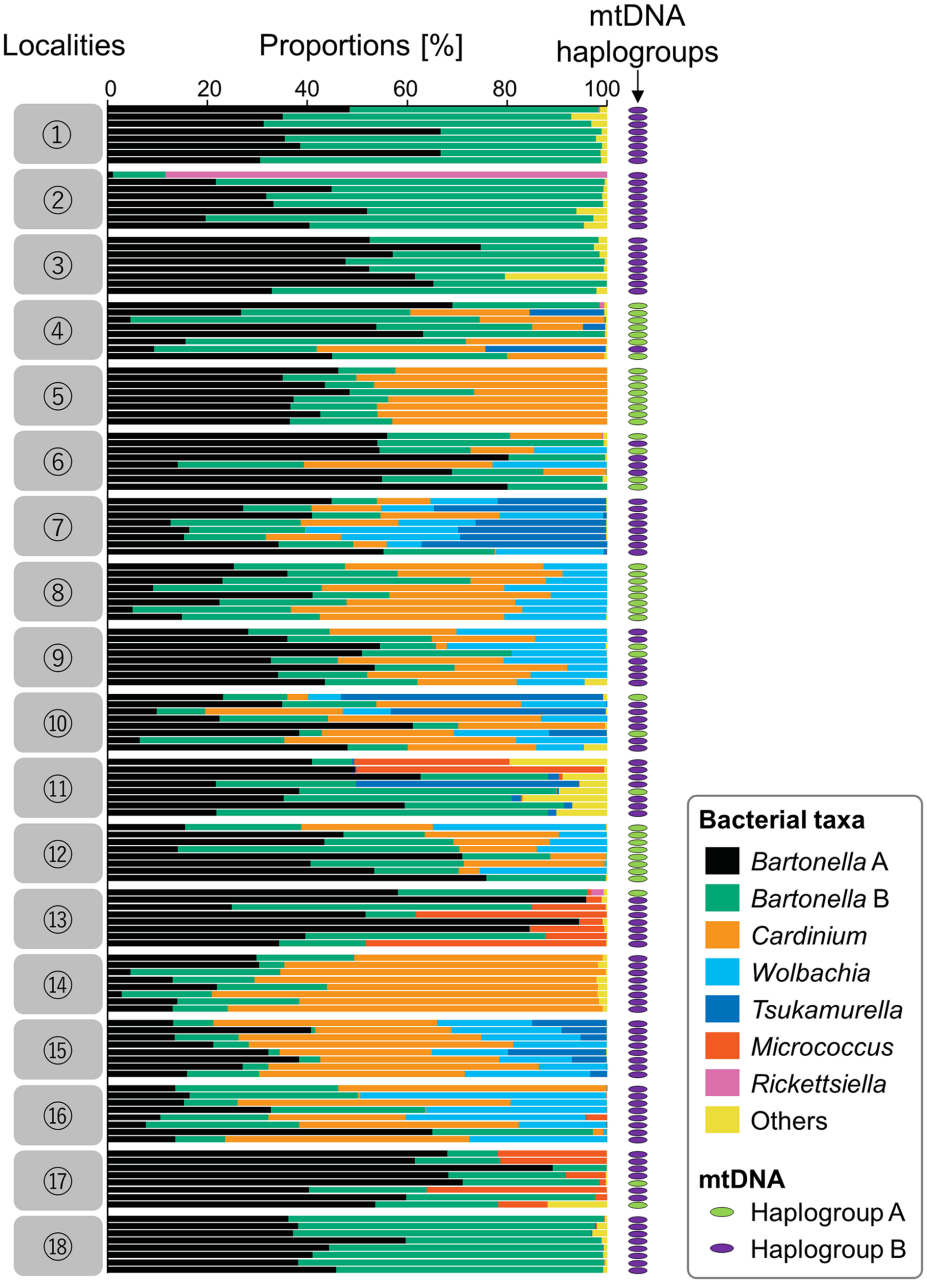
Results of amplicon sequencing of the hypervariable V3/V4 region using 144 individual *D. gallinae* mites from 18 poultry farms (eight individual mites from each poultry farm). For each individual, an mtDNA haplogroup (either A or B) is shown by a colored ellipse at the right (green for haplogroup A; purple for haplogroup B). Assigned bacterial taxa are color-coded as shown in the box on the right. Numbers on the left represent geographic localities shown in Figure 1.

For the collected 144 sample individuals, microbiomes were analyzed through amplicon sequencing of the hypervariable V3/V4 region of 16S rRNA; 8,939,852 reads were reduced into 819 OTU (Supplementary Table 5). The 11 major OTU were *Bartonella* sp. B, *Cardinium*, *Bartonella* sp. A, *Wolbachia*, *Tsukamurella*, *Micrococcus*, *Rickettsiella*, *Staphylococcus*, *Enterobacter*, *Rhodopseudomonas,* and *Psychrobacter* in order of relative frequency (Figure 3; Supplementary Table 6). By assuming that an individual mite has the bacterium when the bacterium represented more than 1% of the tags analyzed, we showed that among the 144 individual mites, all had *Bartonella* sp. A (100%), 140 mites had *Bartonella* sp. B (97.2%), 76 mites had *Cardinium* (52.8%), 52 mites had *Wolbachia* (36.1%), 23 mites had *Tsukamurella* (16.0%), 17 mites had *Micrococcus* (11.8%), and 2 mites had *Rickettsiella* (1.4%). Box plots of the 18 poultry farms were drawn according to the Shannon diversity index (Supplementary Figure 3; Supplementary Table 7).

While *Rickettsia* was previously suggested to be a symbiotic bacterium of *D. gallinae* (De Luna et al., 2009; Moro et al., 2009), the reads of Rickettsiales were not found in the present study, except for *Wolbachia* and a very few reads of plant mitochondria. Additionally, *Spiroplasma*, which was previously detected in a French *D. gallinae* study (Moro et al., 2009), was not found in the present study. A few reads found in some samples were *Erysipelothrix* and *Pasteurella*, which are suspected hen and human pathogens which have been detected in *D. gallinae* (Chirico et al., 2003; Moro et al., 2009; Eriksson et al., 2010; Moro et al., 2011; Hubert et al., 2017). However, the *Erysipelothrix* sequences obtained in this study had only 82–87% similarity with known hen-pathogenic *Erysipelothrix rhusiopathiae* sequences (Takahashi et al., 1994; Accession No.: NR_040837), whereas the *Pasteurella* sequences obtained in this study had 93%–94% similarity with known human opportunistic pathogen *Pasteurella multocida* sequences (Kuhnert et al., 2000; Accession No.: NR_041809). Determining whether these *Erysipelothrix* and *Pasteurella* in *D. gallinae* could be pathogenic for hens and/or humans requires further investigation.

### Phylogenetic analysis of *Bartonella*

**Figure 4:**
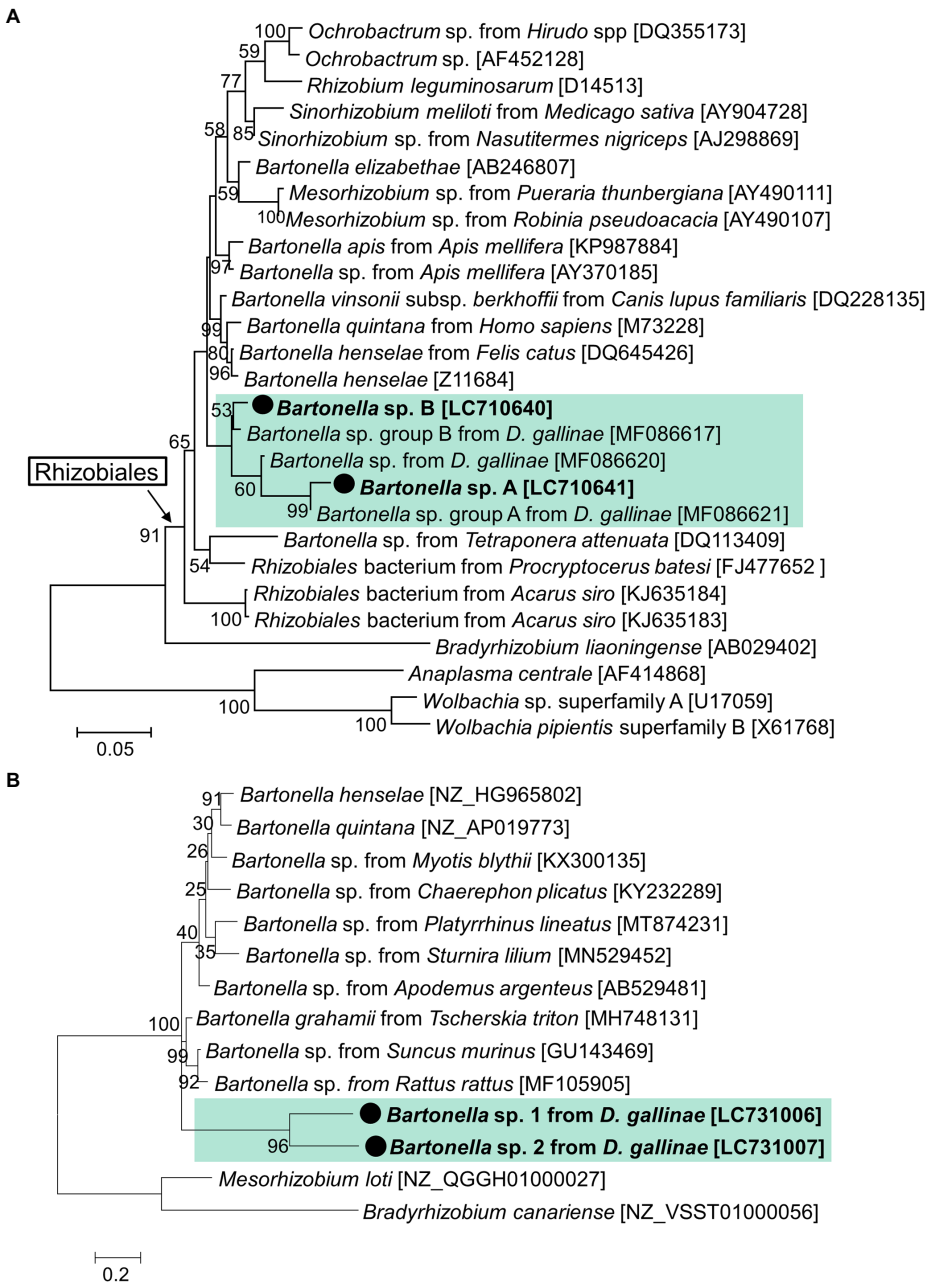
Phylogenetic relationship of the 16S rRNA sequences (A) and rpoB (B) of Bartonella and related species. **(A)** A maximum-likelihood phylogeny (model K2 + G + I) inferred from 1,175 aligned nucleotide sites are shown with bootstrap probability at each node. **(B)** A maximum-likelihood phylogeny (model K2 + G) inferred from 385 aligned nucleotide site. The sequences in bold with black circle are those obtained in this study. OTU of *Bartonella* from *D. gallinae* are shaded with light green. Numbers in square brackets indicate NCBI accession numbers.

The cloning and sequencing of nearly full sequences of 16S rRNA from one sample (8_1) from Tochigi Prefecture identified two strains of *Bartonella*, with each corresponding to either *Bartonella* sp. A or *Bartonella* sp. B. These sequences were closely related but differed in 66 bp among 1,437 bp, and formed a cluster with the *Bartonella* sp. sequences obtained from *D. gallinae* in a previous study (Hubert et al., 2017; Figure 4A). Although not well-supported by bootstrap value, this cluster was distinct from other *Bartonella* and *Rhizobiales* bacteria. It has been reported that 16S rRNA is not a suitable marker for *Bartonella* (La Scola et al., 2003) because of the existence of multiple copies in its genome (Viezens and Arvand, 2008; Banerjee et al., 2020). To distinguish whether *Bartonella* sp. A and *Bartonella* sp. B described by 16S rRNA amplicon sequencing are actually from a single strain, we sequenced a single copy gene *rpoB* (385 bp). An individual 18_4, whose microbiome was mostly occupied by *Bartonella* sp. A and *Bartonella* sp. B with a nearly equal ratio, was subjected to PCR, cloning, and sequencing (Supplementary Figure 4). All the obtained sequences (17 out of 17) were *Bartonella*-like, which consist of two types of sequences differing in 76 bp. This result strongly suggests that *Bartonella* sp. A and *Bartonella* sp. B are distinct strains of *D. gallinae*. Furthermore, these sequences of *rpoB* were a new lineage that differed from the known *Bartonella* and *Rhizobiales* (Figure 4B). It remains unknown, however, which of the *rpoB* sequences corresponds to *Bartonella* sp. A or *Bartonella* sp. B.

### Phylogenetic analysis of *Rickettsiella*

**Figure 5:**
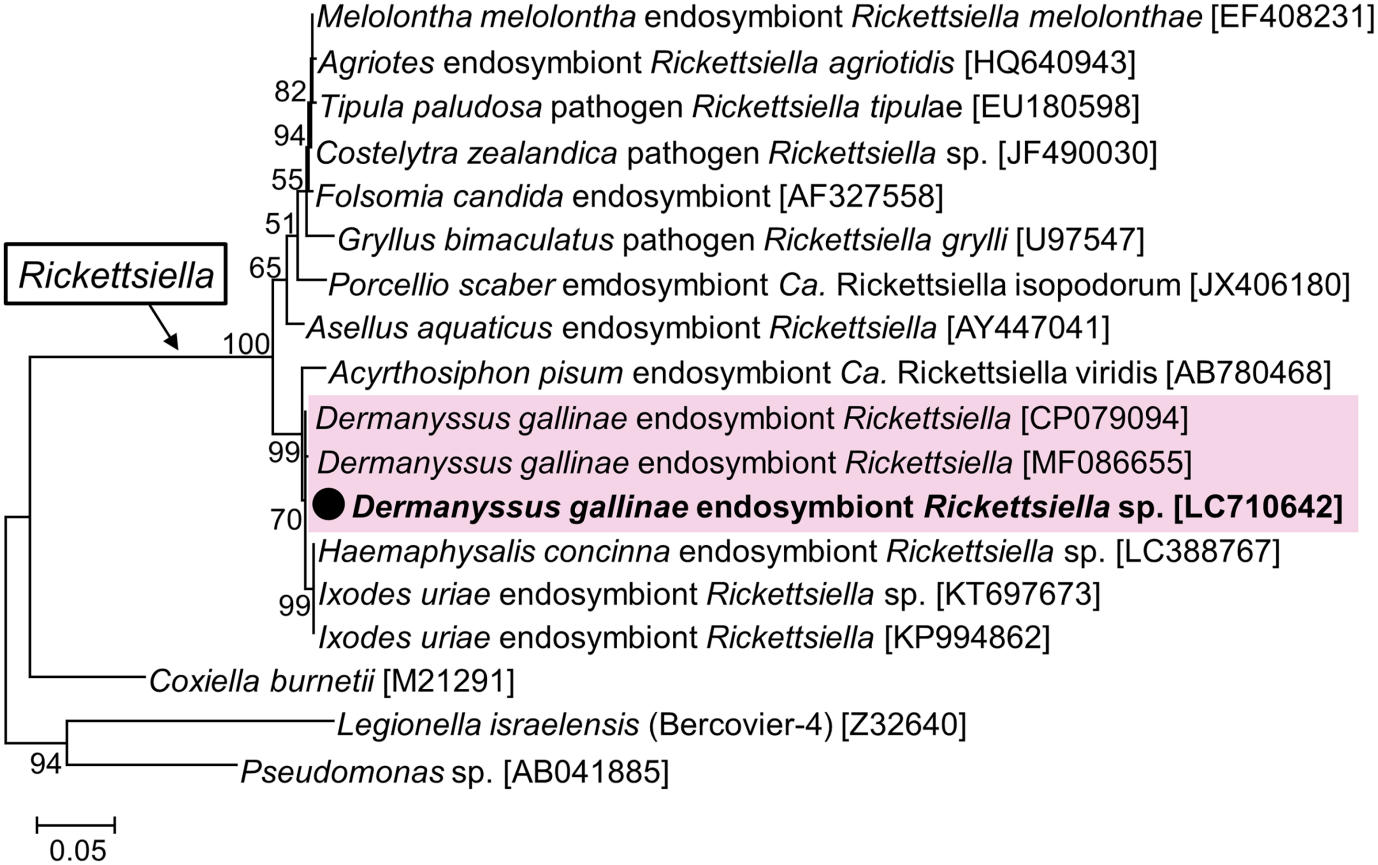
Phylogenetic relationship of the 16S rRNA sequences of *Rickettsiella* and related species. A maximum-likelihood phylogeny (model HKY + G + I) inferred from 1,018 aligned nucleotide sites is shown with bootstrap probability at each node. The sequences in bold with black circle are those obtained in this study. OTU of *Rickettsiella* from *D. gallinae* are shaded in pink. Numbers in square brackets indicate NCBI accession numbers.

A nearly full sequence of *Rickettsiella* 16S rRNA was obtained from a sample from Hokkaido (2_1) which had a relatively high abundance of *Rickettsiella* in the amplicon sequencing (Figure 3). This sequence was identical to the *Rickettsiella* sequence obtained from UK samples, and differed by 1 bp from Czech samples (Figure 5). These *D. gallinae Rickettsiella* sequences were related to *Rickettsiella* which infect the relict tick *Haemaphysalis concinna* and the seabird tick *Ixodes uriae*. The clade composed of the mite/tick *Rickettsiella* sequences also include *Rickettsiella* of the pea aphid *Acyrthosiphon pisum* and is distinct from the other clade composed of *Rickettsiella* from insects and isopods.

### Probable obligate symbionts: *Bartonella* or *Rickettsiella*?

**Figure 6:**
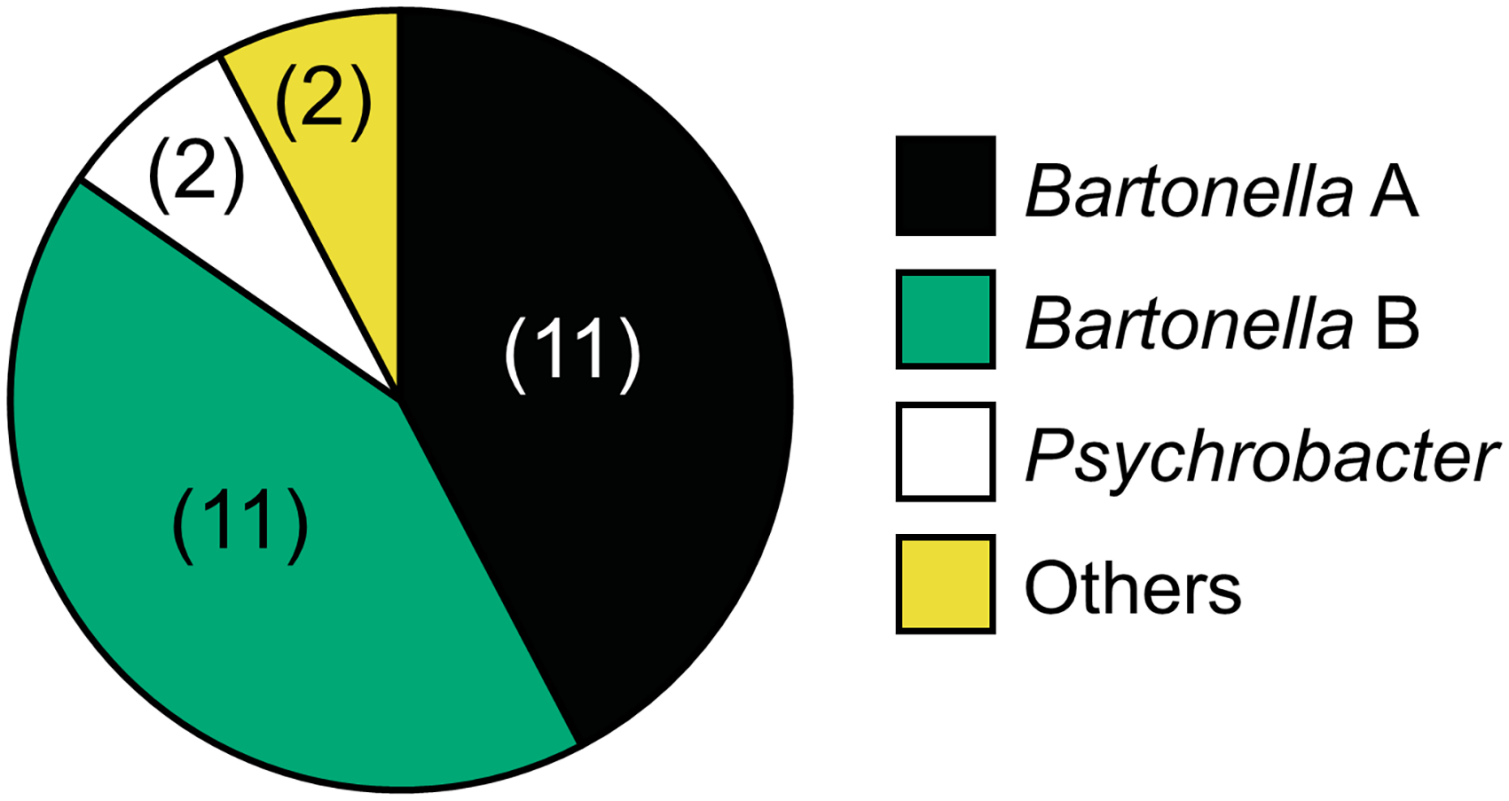
Relative abundance of bacteria associated with *D. gallinae* eggs derived from Ibaraki Prefecture based on 16S rRNA sequences obtained by cloning and sequencing of universal PCR products. The three primary bacterial taxa are colored as shown on the right. Two clones (*Staphylococcus* and *Clostridium*) are indicated with yellow. The number of clones (among 26 clones) is shown in parenthesis.

We hypothesize that *Bartonella* sp. A is necessary for the survival or reproduction of *D. gallinae* because all 144 *D. gallinae* individuals examined had *Bartonella* sp. A. Although, *Bartonella* sp. B might be derived from the blood meal because the 16S rRNA sequence of *Bartonella* sp. B, which was possessed by 140 out of 144 *D. gallinae* individuals (97.2%), matched the sequence of *Bartonella* obtained from sampled avian blood (Accession No. MN320519, MN320520, MN320527). Cloning and sequencing of the bacterial 16S rRNA sequences using surface-sterilized *D. gallinae* eggs from Ibaraki Prefecture (derived from a different farm from those used for amplicon sequencing) identified both *Bartonella* sp. A and B (Figure 6), which is consistent with a previous study which detected *Bartonella* on eggs using amplicon sequencing (Hubert et al., 2017). These results may suggest that both *Bartonella* sp. A and B are vertically transmitted transovarially *via* egg cytoplasm. Then we performed whole-mount FISH targeting bacterial 16S rRNA to examine the localization of symbiotic bacteria using a *D. gallinae* adult and egg collected from Aomori Prefecture. As is shown by microbiome data (population No. 3 in Figure 3), this population has only *Bartonella* sp. A and *Bartonella* sp. B as major bacteria. The FISH preparation showed that, in the adult female, reddish fluorescence is observed throughout the cell cytoplasm except for the nucleus (Supplementary Figure 5), which is typical for intracellular symbionts. The bacteria appear to be present throughout the mite body rather than localized in a distinct bacteriocyte. FISH preparation was also made on eggs, but no clear bacterial image was observed. This may be due to the chorion and/or yolk proteins that inhibit the transmission of fluorescent signals. Note that these results were obtained with the universal 16S rRNA probe for bacteria, so further analysis with *Bartonella*-specific probes would be needed.

Amplicon sequence analysis detected *Rickettsiella* from only 2 out of 144 *D. gallinae* individuals. To test whether *Rickettsiella* was failed to be detected in many samples due to primer mismatches or other factors specific to amplicon sequencing, diagnostic PCR was performed for *Rickettsiella* utilizing primers used in a previous study (Price et al., 2021). Among 16 samples from Hokkaido Prefecture (populations No. 1 and 2), *Rickettsiella* was detected from only one individual (2_1), which is consistent with the results of the amplicon sequence analysis (Supplementary Figure 6); we therefore conclude that *Rickettsiella* infection is very rare in Japanese *D. gallinae*. This contrasts with European populations of *D. gallinae*, which have been reported to be highly infected with *Rickettsiella* (De Luna et al., 2009; Moro et al., 2009; Price et al., 2021). In Czechia, however, *Rickettsiella* was detected from only one of four sample sites studied (Hubert et al., 2017). Although no mitochondrial haplotypes were examined in relation to symbiotic bacteria in the European studies, we cannot rule out the possibility that haplogroups A and B have *Bartonella* as an essential symbiont and haplogroup C has *Rickettsiella* as an essential symbiont. This hypothesis may be worth testing in future research.

To summarize, Japanese *D. gallinae* are predominantly infested with *Bartonella*, which likely plays an important role in the survival of these mites. *Bartonella* bacteria generally have a hemotropic lifestyle and is found in mammalian hosts and blood-sucking parasitic arthropods, such as lice and fleas (Kosoy et al., 2012; Gutiérrez et al., 2014; Theonest et al., 2019). We speculate that *Bartonella* contributes to the synthesis of B vitamins, although this has not yet been demonstrated in any hosts.

As noted in the introduction, *Rickettsiella* is considered to be an important symbiont of *D. gallinae* because it has a nearly complete set of B vitamin synthetic pathways (Price et al., 2021). However, the biotin (vitamin B7) synthesis pathway has been shown to be incomplete due to the loss of bioH (Price et al., 2021). In the present study, two *Rickettsiella*-possessing individuals were found that had both *Bartonella* sp. A and B. Considering the contrasting frequencies of *Bartonella* and *Rickettsiella*, it is likely that *Bartonella* is an important symbiont for *D. gallinae*, whereas *Rickettsiella* has an auxiliary role or pathogenic effect, at least in Japanese red mite populations.

### Facultative symbionts: *Cardinium* and *Wolbachia*

**Figure 7:**
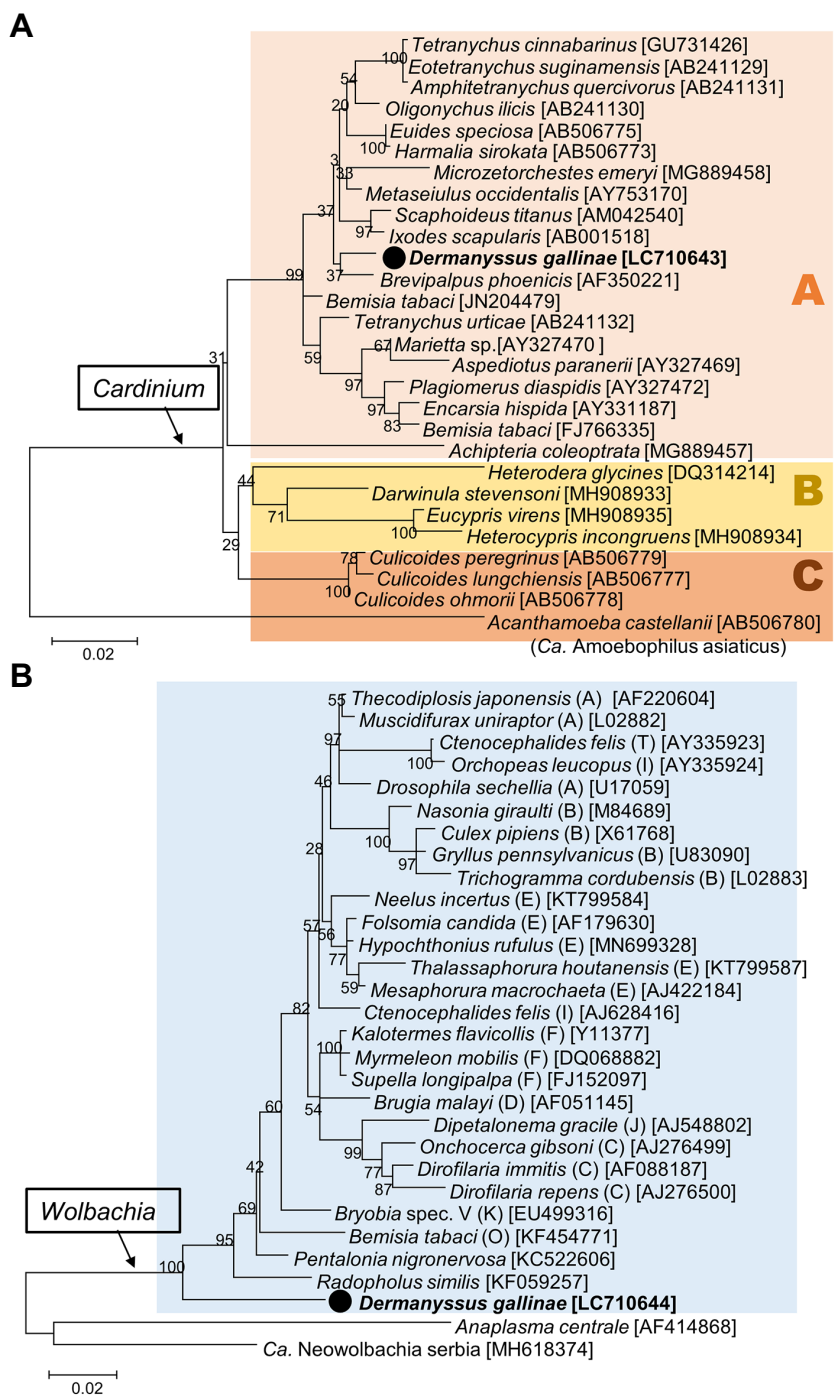
Phylogenetic relationships of the 16S rRNA sequences of *Cardinium* **(A)** and *Wolbachia* **(B)**. **(A)** Maximum-likelihood tree (the model is K2 + G + I based on 1,175 bp) of *Cardinium*, which can be separated into three groups (A, B, and C). **(B)** Maximum-likelihood tree (the model is HKY + G + I based on 1,226 bp) of *Wolbachia*. Supergroups of *Wolbachia* are indicated in round brackets. A bootstrap probability is given at each node. The sequences in bold with black circle are those obtained in this study. Numbers in square brackets indicate NCBI accession numbers.

The nearly full sequences of 16S rRNA of *Cardinium* and *Wolbachia* were obtained from one sample (8_1) from Tochigi Prefecture. Phylogenetically, the *Cardinium* derived from *D. gallinae* fell into the group A of *Cardinium*, the most common group found among various arthropods (Nakamura et al., 2009) (Figure 7A). In contrast, the *Wolbachia* obtained from *D. gallinae* was idiosyncratic, and was located basally of all previously published *Wolbachia*, but formed a well-supported clade with other *Wolbachia* (Figure 7B).

Both *Cardinium* and *Wolbachia* are known as maternally inherited symbionts which manipulate host reproduction in various manners (such as cytoplasmic incompatibility, feminization, male killing, and induction of parthenogenesis) to enhance their own transmission (Werren et al., 2008; Hurst and Frost, 2015). In spider mites, it has been reported that *Wolbachia* and *Cardinium* cause cytoplasmic incompatibility (Breeuwer, 1997; Gotoh et al., 2007), and that *Cardinium* causes feminization (Weeks et al., 2001; Chigira and Miura, 2005). Because of the linkage disequilibrium between maternally inherited bacteria and host mitochondrial DNA, the spread of maternally inherited symbionts can result in indirect selection on mitochondrial DNA (Hurst and Jiggins, 2005). This study compared the infection frequencies of *Cardinium* and *Wolbachia* in respective mtDNA haplotypes (Supplementary Figure 7). While haplogroup BJ1 had a low *Cardinum* and/or *Wolbachia* infection rate (infection rate of either bacterium is 25.4% (18/71)), haplogroup AJ1 and haplogroup BJ2 had very high infection rates of 79.1% (34/43) and 96.7% (29/30), respectively. These conspicuously biased infection rates may indicate that *Cardinum* and/or *Wolbachia* manipulate host reproduction. The LBF that inferred dependency between *Cardinium* and *Wolbachia* was 26.539, which is very strong evidence for correlation (Gilks et al., 1995). We hypothesize that *Wolbachia*, *Cardinium*, or both may induce reproductive manipulations in *D. gallinae* and its symbionts, and that the associated cytoplasms (i.e., mitochondrial haplotypes) spread in the host populations.

## Conclusion

We investigated the microbiome of the red poultry mite *D. gallinae* in Japanese populations. Using Illumina Miseq amplicon sequencing, and full 16S rRNA sequencing using universal primers, we identified the following properties of the *D. gallinae* microbiome. First, all individual mites had *Bartonella* sp. A, and most individuals had *Bartonella* sp. B, which are both closely related to the *Bartonella* harbored by *D. gallinae* in Czechia. These *Bartonella* species, which are probably vertically transmitted through eggs, may play important roles in *D. gallinae* survival. Second, unlike many European populations, *Rickettsiella* was rarely found and is unlikely to play an important role in the survival of *D. gallinae*, at least in Japanese populations. Third, *Cardinium* and *Wolbachia* were found at relatively high frequencies, and while the *Cardinium* identified belonged to a lineage commonly found in insects and other arthropods, the *Wolbachia* belonged to a novel lineage that is located basally to all other *Wolbachia* found so far. It is possible that these *Cardinium* and *Wolbachia* strongly impact the mitochondrial genome dynamics of *D. gallinae*. It should also be noted that regarding all the detected bacteria, no direct evidence for their roles within *D. gallinae* was identified because *D. gallinae* could not be successfully reared despite multiple attempts following Bruneau et al. (2001). Inoculation and removal of each bacterium to fulfill Koch’s postulate, as well as genome sequencing of each bacterium will be necessary to evaluate the impact of the bacteria on *D. gallinae*.

## Data availability statement

The data presented in the study are deposited in NCBI, accession numbers LC710609-LC710644, LC731006-LC731007, and DRR376882–DRR377025.

## Author contributions

YN, TS, KW, HE, and DK conceived and designed the experiments. YN organized all the samples and performed experiments. TS analyzed the raw reads of Illumina Miseq. YN wrote the draft manuscript and DK revised the manuscript with the inputs from all authors. All authors contributed to the article and approved the submitted version.

## Funding

The authors declare that this study received funding from SC Environmental Science Co., Ltd. The funder was not involved in the study design, collection, analysis, interpretation of data, the writing of this article, or the decision to submit it for publication.

## Conflict of interest

HE was employed by the company SC Environmental Science Co., Ltd.

The remaining authors declare that the research was conducted in the absence of any commercial or financial relationships that could be construed as a potential conflict of interest.

## Publisher’s note

All claims expressed in this article are solely those of the authors and do not necessarily represent those of their affiliated organizations, or those of the publisher, the editors and the reviewers. Any product that may be evaluated in this article, or claim that may be made by its manufacturer, is not guaranteed or endorsed by the publisher.

## Footnotes

^1^ https://data.qiime2.org/2022.2/common/silva-138-99-515-806-nb-classifier.qza

^2^ https://data.qiime2.org/2022.2/common/gg-13-8-99-515-806-nb-classifier.qza

^3^ https://view.qiime2.org/

^4^ http://www.evolution.reading.ac.uk/BayesTraitsV4.0.0/BayesTraitsV4.0.0.html
