# Supplementary figures and images for: Genetic variations and microbiome of the poultry red mite Dermanyssus gallinae
Source: Front Microbiol. 2022 Nov 8;13:1031535. doi: 10.3389/fmicb.2022.1031535 (PMC9680903; doi:10.3389/fmicb.2022.1031535)

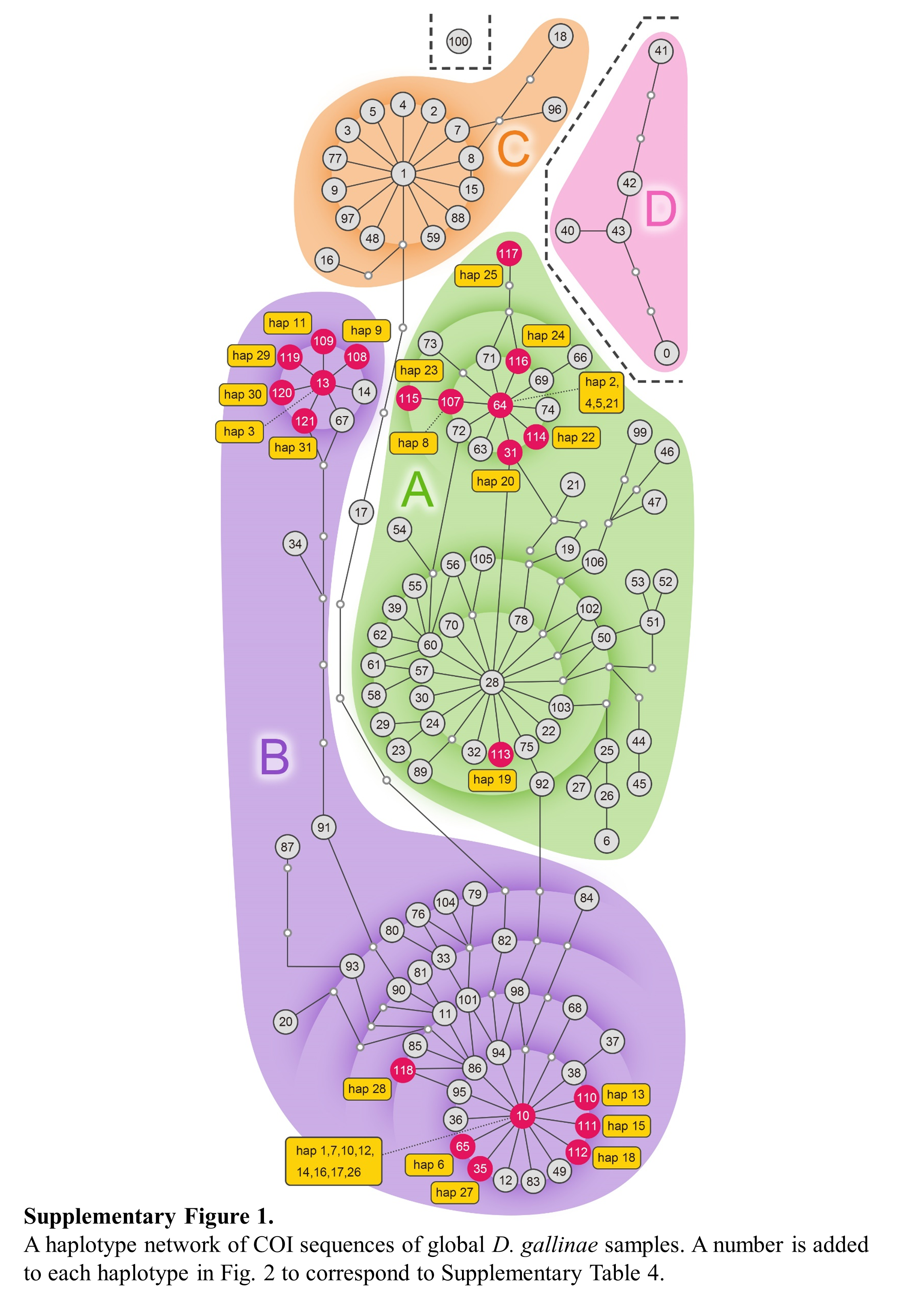

Supplement: Supplementary file 2 [file Image_1.TIF]

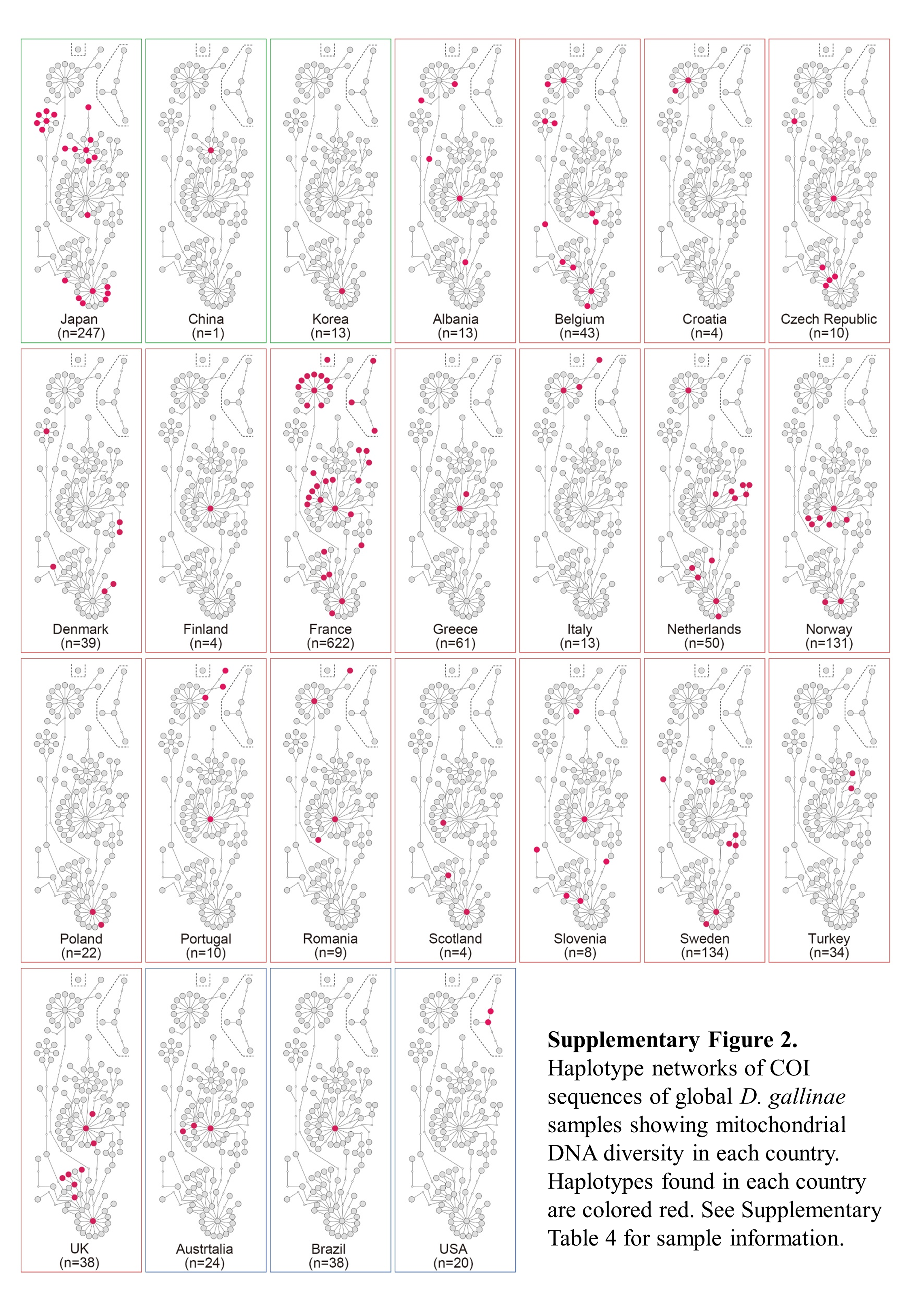

Supplement: Supplementary file 3 [file Image_2.TIF]

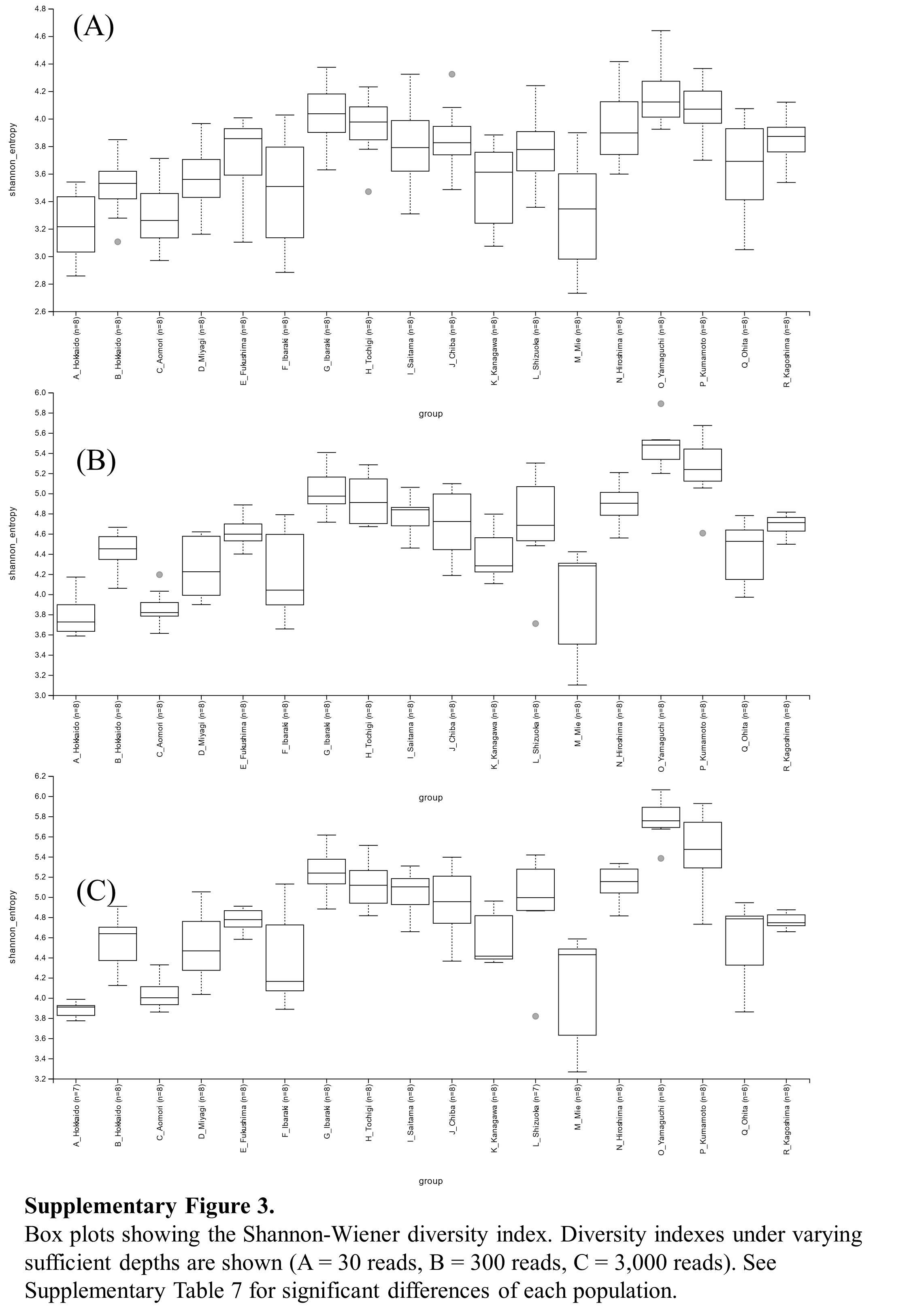

Supplement: Supplementary file 4 [file Image_3.TIF]

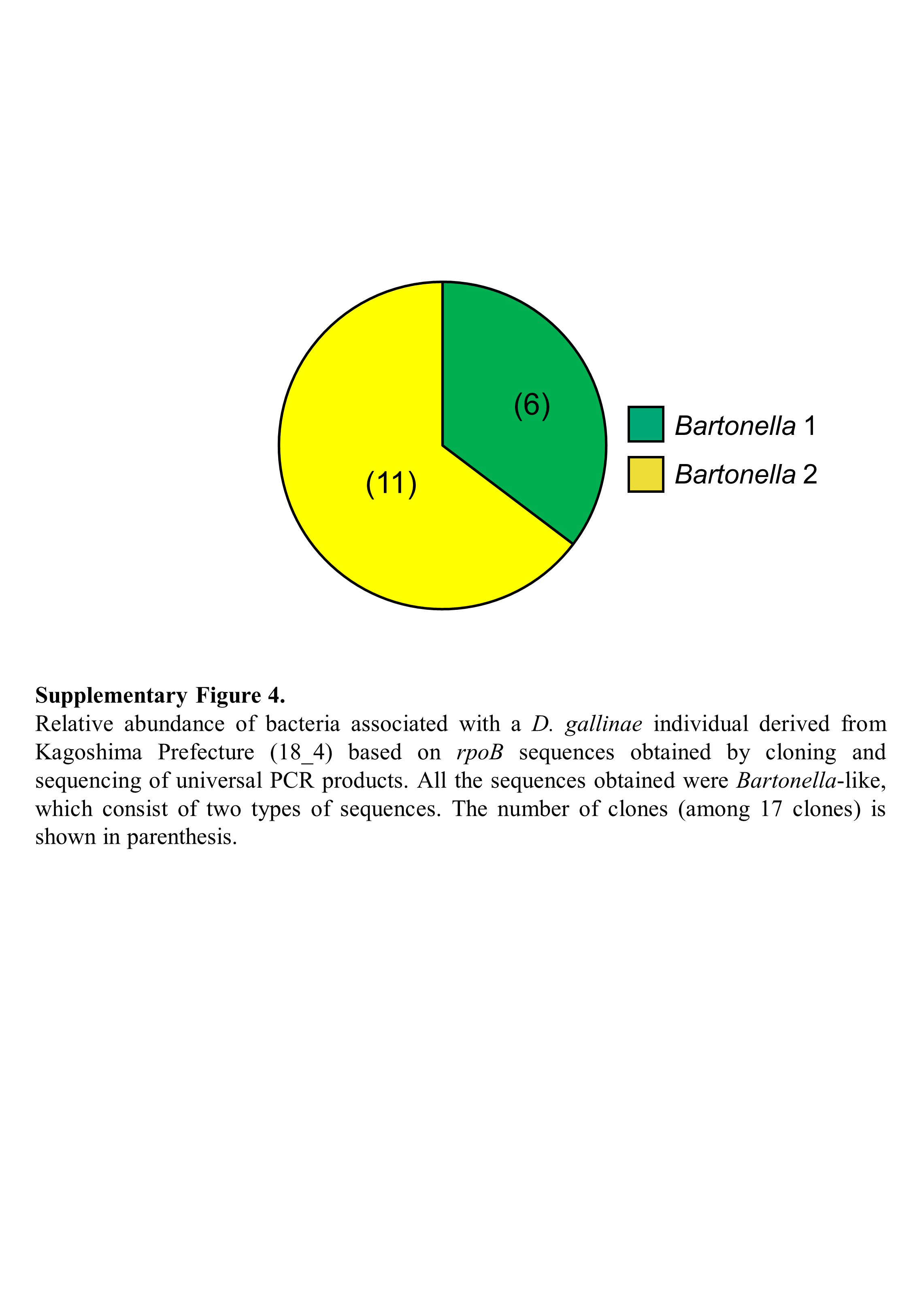

Supplement: Supplementary file 5 [file Image_4.TIF]

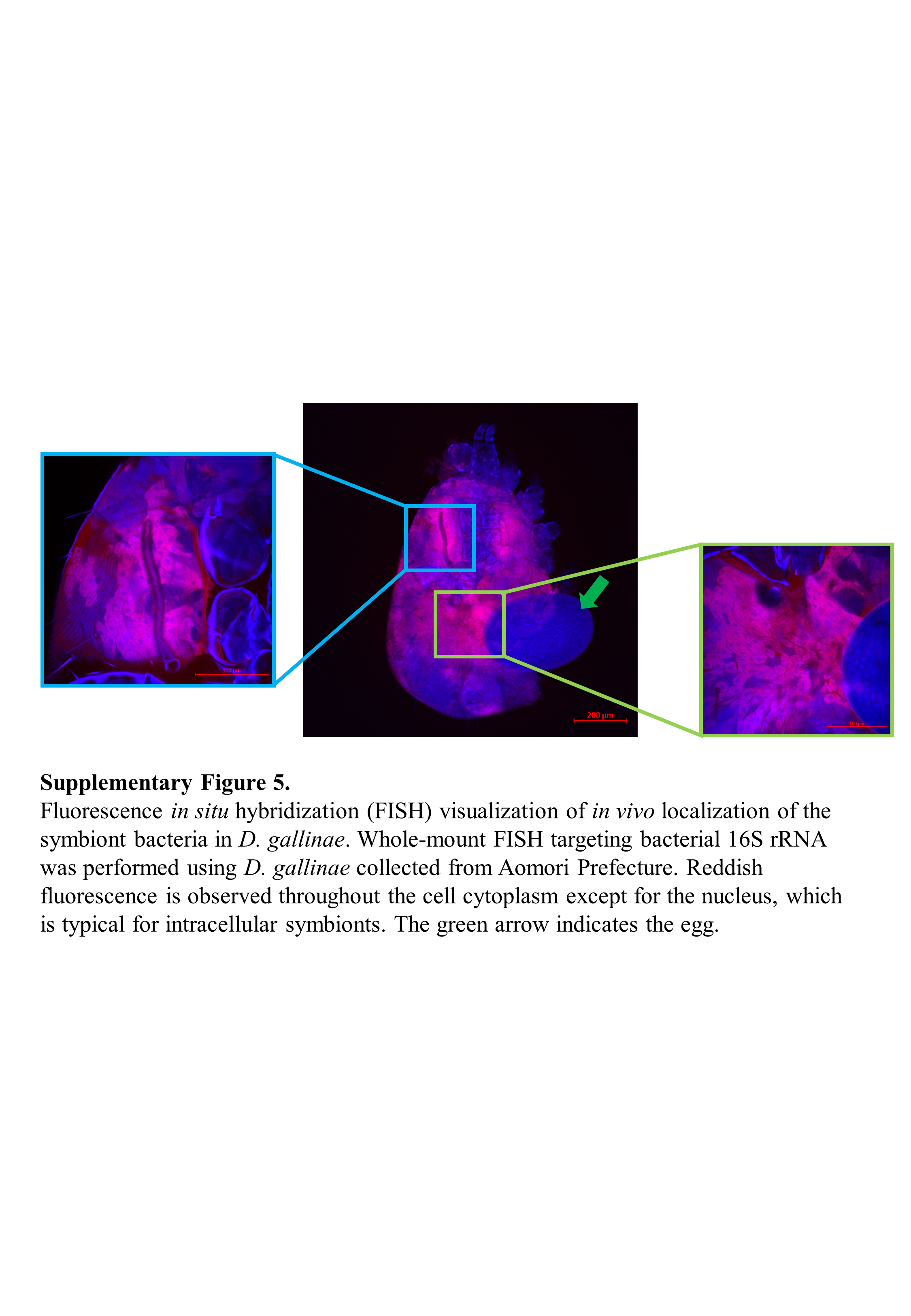

Supplement: Supplementary file 6 [file Image_5.TIF]

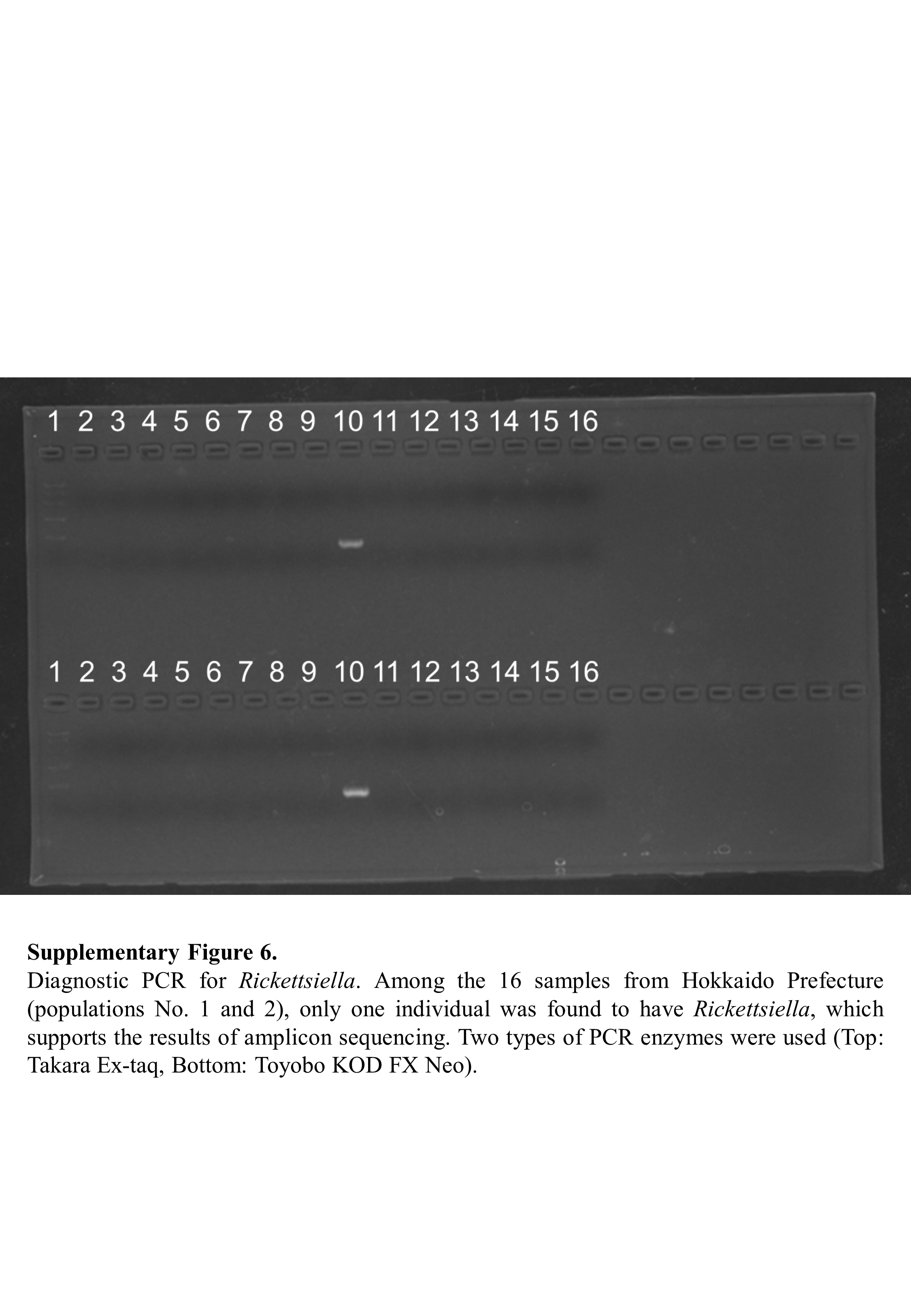

Supplement: Supplementary file 7 [file Image_6.TIF]

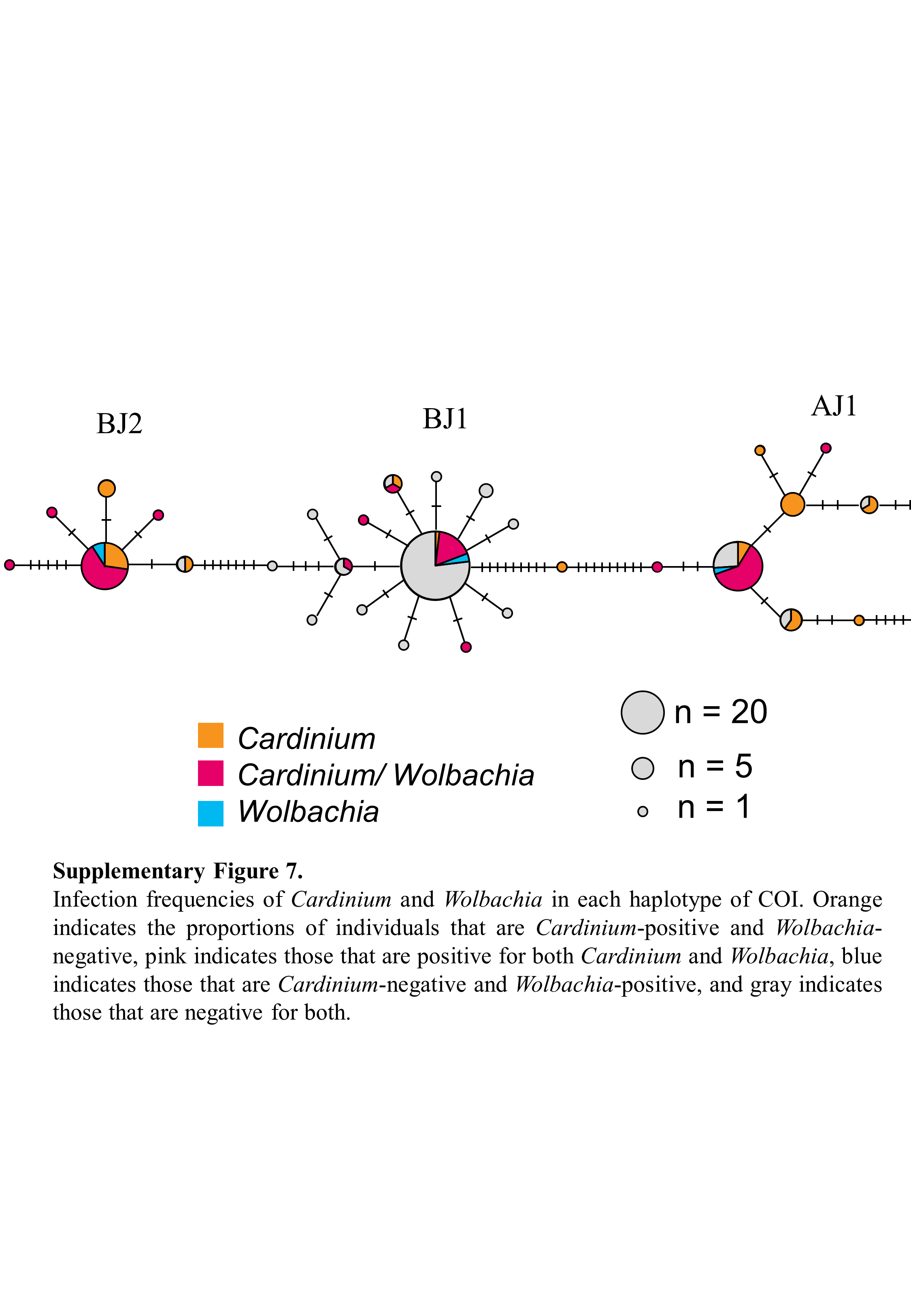

Supplement: Supplementary file 8 [file Image_7.TIF]
